# TIGIT signaling and its influence on T cell metabolism and immune cell function in the tumor microenvironment

**DOI:** 10.3389/fonc.2023.1060112

**Published:** 2023-02-17

**Authors:** Nouria Jantz-Naeem, Romy Böttcher-Loschinski, Katrin Borucki, Marisa Mitchell-Flack, Martin Böttcher, Burkhart Schraven, Dimitrios Mougiakakos, Sascha Kahlfuss

**Affiliations:** ^1^ Institute of Molecular and Clinical Immunology, Medical Faculty, Otto-von-Guericke University Magdeburg, Magdeburg, Germany; ^2^ Department of Hematology and Oncology, University Hospital Magdeburg, Otto-von-Guericke University Magdeburg, Magdeburg, Germany; ^3^ Institute of Clinical Chemistry, Department of Pathobiochemistry, Medical Faculty, Otto-von-Guericke University Magdeburg, Magdeburg, Germany; ^4^ Department of Oncology, The Bloomberg~Kimmel Institute for Cancer Immunotherapy, Johns Hopkins University School of Medicine, Baltimore, MD, United States; ^5^ Health Campus Immunology, Infectiology and Inflammation (GCI), Medical Faculty, Otto-von-Guericke University Magdeburg, Magdeburg, Germany; ^6^ Institute of Medical Microbiology and Hospital Hygiene, Medical Faculty, Otto-von-Guericke University Magdeburg, Magdeburg, Germany; ^7^ Center for Health and Medical Prevention (CHaMP), Otto-von-Guericke-University, Magdeburg, Germany

**Keywords:** T cells, metabolism, cancer, therapy, microenviroment

## Abstract

One of the key challenges for successful cancer therapy is the capacity of tumors to evade immune surveillance. Tumor immune evasion can be accomplished through the induction of T cell exhaustion *via* the activation of various immune checkpoint molecules. The most prominent examples of immune checkpoints are PD-1 and CTLA-4. Meanwhile, several other immune checkpoint molecules have since been identified. One of these is the T cell immunoglobulin and ITIM domain (TIGIT), which was first described in 2009. Interestingly, many studies have established a synergistic reciprocity between TIGIT and PD-1. TIGIT has also been described to interfere with the energy metabolism of T cells and thereby affect adaptive anti-tumor immunity. In this context, recent studies have reported a link between TIGIT and the hypoxia-inducible factor 1-α (HIF1-α), a master transcription factor sensing hypoxia in several tissues including tumors that among others regulates the expression of metabolically relevant genes. Furthermore, distinct cancer types were shown to inhibit glucose uptake and effector function by inducing TIGIT expression in CD8^+^ T cells, resulting in an impaired anti-tumor immunity. In addition, TIGIT was associated with adenosine receptor signaling in T cells and the kynurenine pathway in tumor cells, both altering the tumor microenvironment and T cell-mediated immunity against tumors. Here, we review the most recent literature on the reciprocal interaction of TIGIT and T cell metabolism and specifically how TIGIT affects anti-tumor immunity. We believe understanding this interaction may pave the way for improved immunotherapy to treat cancer.

## 1 Introduction

### 1.1 Reciprocal metabolic interaction of tumor cells and T cells within the tumor microenvironment

Tumors are notorious for evading surveillance of the immune system *via* T cell hyporesponsiveness and dysfunction (1, 2). In particular, limited nutrient availability, in particular the scarcity of glucose (3) and tryptophan (4, 5) which are required for normal cell functionality, in the tumor microenvironment (TME) due to competition can impair CD8^+^ cytotoxic T cells (CTL) proliferation, survival, and effector function (4–6). In this context, tumor cells have been shown to express the enzyme indoleamine 2,3-dioxygenase (IDO), which on the one hand depletes tryptophan, a critical amino acid needed for T cell proliferation (4, 5), and on the other hand produces kynurenine, a T cell suppressive metabolic ‘waste’ product (7). It is noteworthy that the role of effector CD4^+^ T cells during anti-tumor immunity is not as well resolved as it is for CD8^+^ T cells (8). In addition, hypoxia within the TME can diminish anti-tumor activity directly by inhibiting NK cell-mediated killing (9), or by inducing T cell apoptosis through inhibition of CCR7 expression *via* the A2A receptor signaling pathway (10). Hypoxia has also been demonstrated to upregulate immune checkpoint proteins such as PD-L1 on tumor cells (11–13). Additionally, metabolites produced by tumor cells can promote tumor immune evasion. In this regard, adenosine, a byproduct of the enzymatic breakdown of adenosine 5’-triphosphate (ATP) *via* the ectonucleotidases CD39 and CD73, promotes tumor growth, survival, and metastasis and also impairs CD8^+^ T cell signaling and function (14–18). Furthermore, acidification of the TME through the generation of lactic acid by the tumor itself impairs respiration, chemotaxis, and cytokine production of CTLs (6, 19). Altogether, the TME is a unique metabolic niche that consists of several mechanisms to escape immune surveillance by impairing T cell metabolism and effector function.

### 1.2 The role of T cell and tumor cell metabolism for anti-tumor immunity

For T cells to be able to undergo essential processes such as proliferation, growth and differentiation, they need to metabolically adapt to their new requirements, a process also referred to as metabolic reprogramming (20, 21). Naïve T cells mainly make use of fatty acid oxidation, while activated T cells tend to shift from the energetically more favorable oxidative phosphorylation (OXPHOS) to the Warburg metabolism (22–24) to fulfill their need for various metabolic resources. In order to facilitate this kind of metabolic reprogramming during T cell activation, several different signaling cascades and transcription factors come into play. IL-2, a classical growth factor cytokine, and the ligation of costimulatory proteins will enable the metabolic transition to glycolysis by increasing the expression of nutrient transporters and activation of mTOR, a key metabolic regulator (25–27). Together with c-Myc, a protein that activates the transcription of metabolic genes essential for T cell activation (28), mTOR induces the increased expression of glucose transporter 1 (GLUT1) and CD98, a protein responsible for transporting amino acids into the cell (29). To summarize, it can be stated that the metabolic profile of T cells will determine their functional state.

There is increasing appreciation for the fact that a metabolic interplay between tumor and immune cells exists in the TME (30, 31). Further, there is evidence that immune checkpoint proteins themselves have an effect on T cell metabolism, reviewed comprehensively by Lim et al. (32). Kleffel et al. (33) have demonstrated that melanoma cell intrinsically expressed PD-1 upregulates the Akt/mTOR signaling pathway in cancer cells. In another study by Chang et al. (31), tumor PD-L1 expression promoted glycolysis and the activation of Akt/mTOR in tumor cells, while simultaneously suppressing the activity of mTOR in T cells by competing for glucose. The blocking of PD-L1, PD-1 and CTLA-4 resulted in altered concentrations of extracellular glucose (31). This is noteworthy as acidosis in the TME can limit the anti-tumor activity of CTL, as well as suppress their proliferation and cytokine production (34). It is plausible to assume that several immune checkpoint proteins can promote glycolysis in tumor cells, therefore creating a nutrient competitive scenario between tumor cells and immune cells within the TME.

### 1.3 Immune checkpoints in T cell immunity

To elicit a successful immune response against tumors, T cells need to become fully activated. This activation depends on two distinct signals. The first signal represents the engagement of the T cell receptor (TCR) by cognate peptide:MHC class I or II complexes (pMHC) presented by antigen presenting cells (APCs) (35). The second signal involves the co-stimulation *via* B7 proteins on APCs that interact with cluster of differentiation (CD)28 expressed on the surface of T cells (36, 37). Unchecked and/or persistent activation of T cells could lead to aberrant inflammation causing severe damage to host tissue. Because of this, it is necessary that T cell activation is closely regulated by co-stimulatory and co-inhibitory proteins, referred to as immune checkpoints (38).

**Figure 1:**
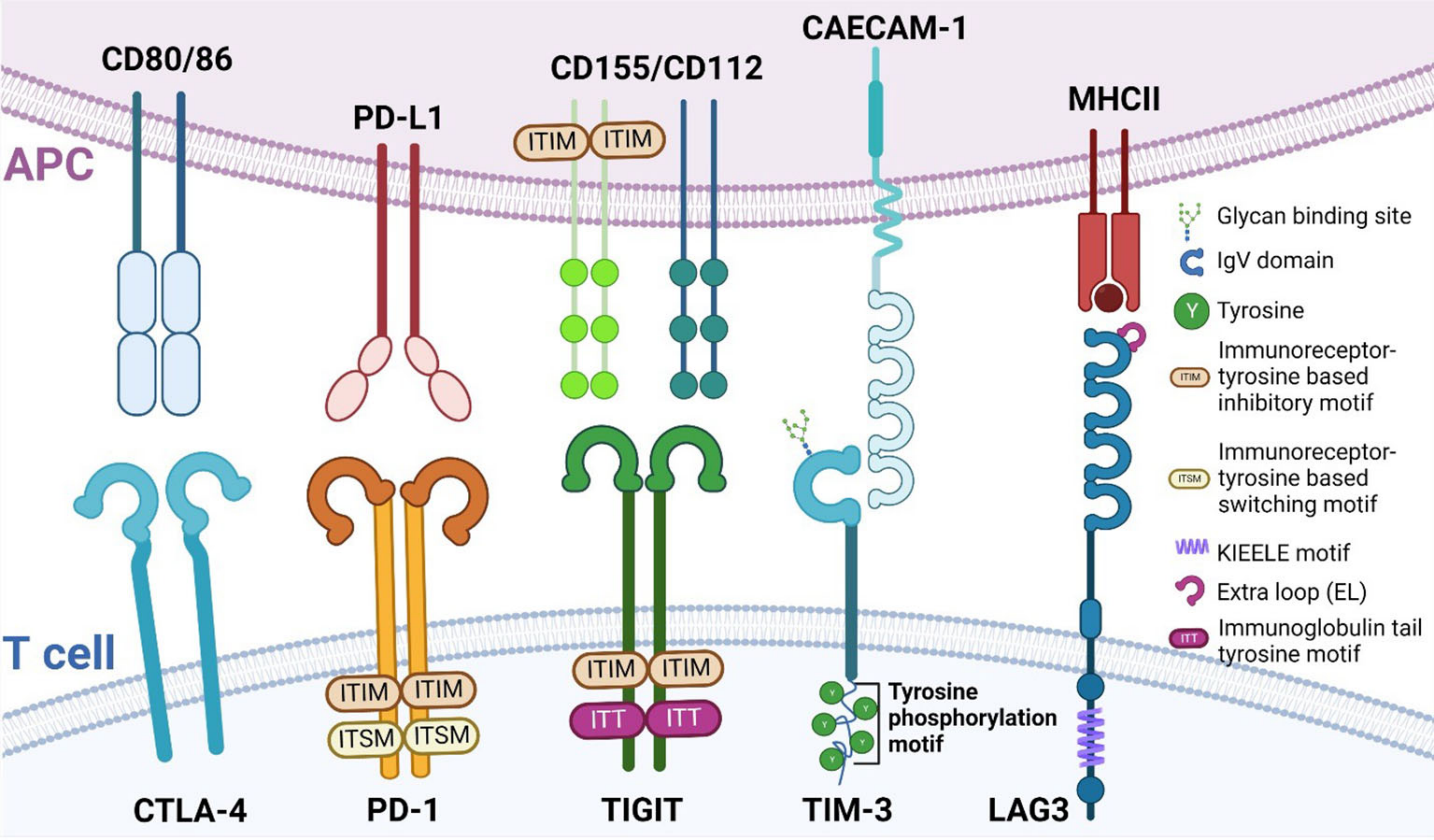
Structure of different immune checkpoints. Immune checkpoints and their structure expressed on T cells (bottom) and their respective ligands expressed on APCs (top). Depicted here are CTLA-4, PD-1, TIGIT, TIM-3 and LAG3. APC, antigen presenting cell; CTLA-4, Cytotoxic T lymphocyte antigen 4; PD-1, Programmed Death-1; TIGIT, T cell immunoglobulin and ITIM domain; TIM-3, T cell immunoglobulin domain and mucin domain 3; LAG3, lymphocyte activation gene-3.

In the past 10 years, the development of novel immunotherapies has been enormously successful especially within the areas of chimeric antigen receptor (CAR) T cells (39), bispecific antibodies capable of binding two targets simultaneously (40), and immune checkpoint inhibitors (ICI) (41). However, despite the enormous success of ICIs, many patients show or acquire resistance to treatment with ICIs (42). Consequently, the latter has resulted in a need for the identification of novel immune checkpoints such as lymphocyte activation gene-3 (LAG3) (43), V-domain Ig suppressor of T cell activation (VISTA) (44), B and T cell attenuator (BTLA) (45), B7 homolog 3 protein (B7-H3) (46), T cell immunoglobulin and mucin-domain containing-3 (TIM3) (47) and T cell immunoglobulin and ITIM domain (TIGIT) (48). These proteins each have distinct ligands and suppress T cell function through several mechanisms to ensure there is proper regulation of the T cell response. In the following paragraphs, we will briefly introduce several immune checkpoints by structure and function. **Figure 1** details these structural differences and similarities between the different immune checkpoints.

#### 1.3.1 PD-1

Programmed Death-1 (PD-1) is a type I transmembrane protein that is expressed in several immune cells, such as T, B, and NK cells. Structurally, it is composed of an extracellular immunoglobulin-like binding domain, a transmembrane region and a cytoplasmic domain containing an immunoreceptor tyrosine-based inhibitory motif (ITIM) and an immunoreceptor tyrosine-base switch motif (ITSM) (49). Engagement of PD-L1 with its receptor results in T cell dysfunction, exhaustion, and production of the immunosuppressive cytokine IL10 within the tumor (50). With the FDA approval for Nivolumab and Pembrolizumab, the potential of blocking PD-1 was realized and successfully applied to improve patient outcomes.

#### 1.3.2 CTLA-4

Cytotoxic T lymphocyte antigen 4 (CTLA-4), also known as CD152, and CD28 are homologous receptors expressed on T cells. While structurally similar, they mediate opposing functions in T cell activation (51–54). Blockade of CTLA-4, such as with Ipilimumab (55), results in the amelioration of the immune response against tumors.

#### 1.3.3 LAG3

LAG3 and CD4 share very similar structures in that they both have four extracellular Ig-like domains (56, 57). Interestingly, LAG3 has a 100-fold higher binding affinity with MHC class II (MHCII) compared to CD4, which is why MHCII is presumed to be the ligand for LAG3 (43) and why LAG3 may be a negative competitor of CD4 (58–62).

#### 1.3.4 TIM3

Contrary to other immune checkpoint proteins, TIM3 does not consist of classical inhibitory signaling motifs such as ITIMS, but instead contains five conserved tyrosine residues (47). Two of these residues can be phosphorylated by Src kinases and are essential for downstream signal transduction (63, 64). Thus far, four distinct ligands, both soluble and surface-bound, have been found to interact with the IgV domain of TIM3 (phosphatidylserine (PtdSer), high-mobility group box-1 protein (HMGB1), carcinoembryonic antigen-related cell adhesion molecule 1 (CAECAM-1) and galectin-9 (Gal-9)) (65). It is noteworthy to mention that PD-1 and TIM-3 can share ligands, as is the case with Gal-9 (66). Tumor-infiltrating dendritic cells (DC) highly express TIM3, which can compete with nucleic acid binding to its ligand high-mobility group protein B1 (HMGB1), reducing anti-tumor immunity otherwise mediated by nucleic acids (67). TIM3 also works to inhibit T cells *via* interaction with the ligand Caecam1 (68).

#### 1.3.5 TIGIT

T cell immunoglobulin and ITIM domain (TIGIT) was first identified in 2009 as an inhibitory immune checkpoint by Yu et al. (48). TIGIT has an extracellular immunoglobulin variable region, a transmembrane domain, as well as a cytoplasmic portion that contains an ITIM and an immunoglobulin tail tyrosine (ITT)-like phosphorylation motif (48), by which it delivers its inhibitory signals. TIGIT expression is restricted to lymphocyte and is found mainly on memory T cells and regulatory T cells (Tregs) as well as on NK cells (48, 69). Niebel et al. (70) have suggested that the expression of TIGIT mRNA is regulated *via* the methylation of the *TIGIT* gene. TIGIT binds to poliovirus receptor (PVR), also known as CD155 (71) with the highest binding affinity, as well as PVR ligand (PVRL) 2, also known as CD112 or Nectin-2 and PVRL3, also known as CD113 or Nectin-3 with lower affinity (71). Similar to CTLA-4/B7/CD28 pathway (72), TIGIT achieves its inhibitory effects by competing with other ligands such as CD266 or CD96 (73). The hypothesis that TIGIT inhibits T cell proliferation has been tested by several groups (74–76) and they reported a direct inhibitory effect.

Concerning the immunosuppressive effect of TIGIT, several mechanisms may explain its function. Among them, TIGIT signaling has been shown to inhibit NK cell degranulation and cytotoxicity (69, 77), where Stanietsky et al. (69) have demonstrated that this inhibitory effect is mediated directly *via* the ITIM of TIGIT. Additionally, TIGIT prevents CD226 signaling in T cells by preventing the homodimerization of the protein (78). CD226 transmits an activating signal and consequently induces the aggregation of LFA-1, an important integrin involved in T cell migration as well as cytotoxicity (79), where aggregation of integrins affects their conformation and the interaction with their ligand (80). The Treg response has also been reported to be modulated by TIGIT (78, 81). In these studies, TIGIT^+^ Tregs express higher levels of classical Treg genes, such as the transcription factor forkhead box P3 (FoxP3), and the surface molecules CD25 and CTLA-4. The engagement of TIGIT further leads to the secretion of IL10, a hallmark immunosuppressive cytokine, which selectively dampens T helper (Th)1 and Th17 immune responses (78). In certain types of cancer such as follicular lymphoma, TIGIT is strongly expressed by intratumoral Tregs as well as memory CD8^+^ T cells. Here, high numbers of TIGIT-expressing tumor infiltrating lymphocytes have been correlated with a poor survival rate (82). As such, TIGIT may in the future be used as a prognostic marker, since elevated expression in T and NK cells predicts negative clinical outcomes (83–90). Based on these findings, TIGIT has become the subject of increased research as a target for cancer therapy, especially in combination with other ICIs, such as PD-1 inhibitors (91).

We here set out to review the literature of the past 20 years on the reciprocal interaction of TIGIT and the T cell metabolism, how it affects anti-tumor immunity, and how a better understanding of this interaction can pave the way for improved immunotherapy to treat cancer.

## 2 Main review

### 2.1 Interaction of TIGIT and the metabolic TME

#### 2.1.1 Inhibition of glucose metabolism in T cells

**Figure 2:**
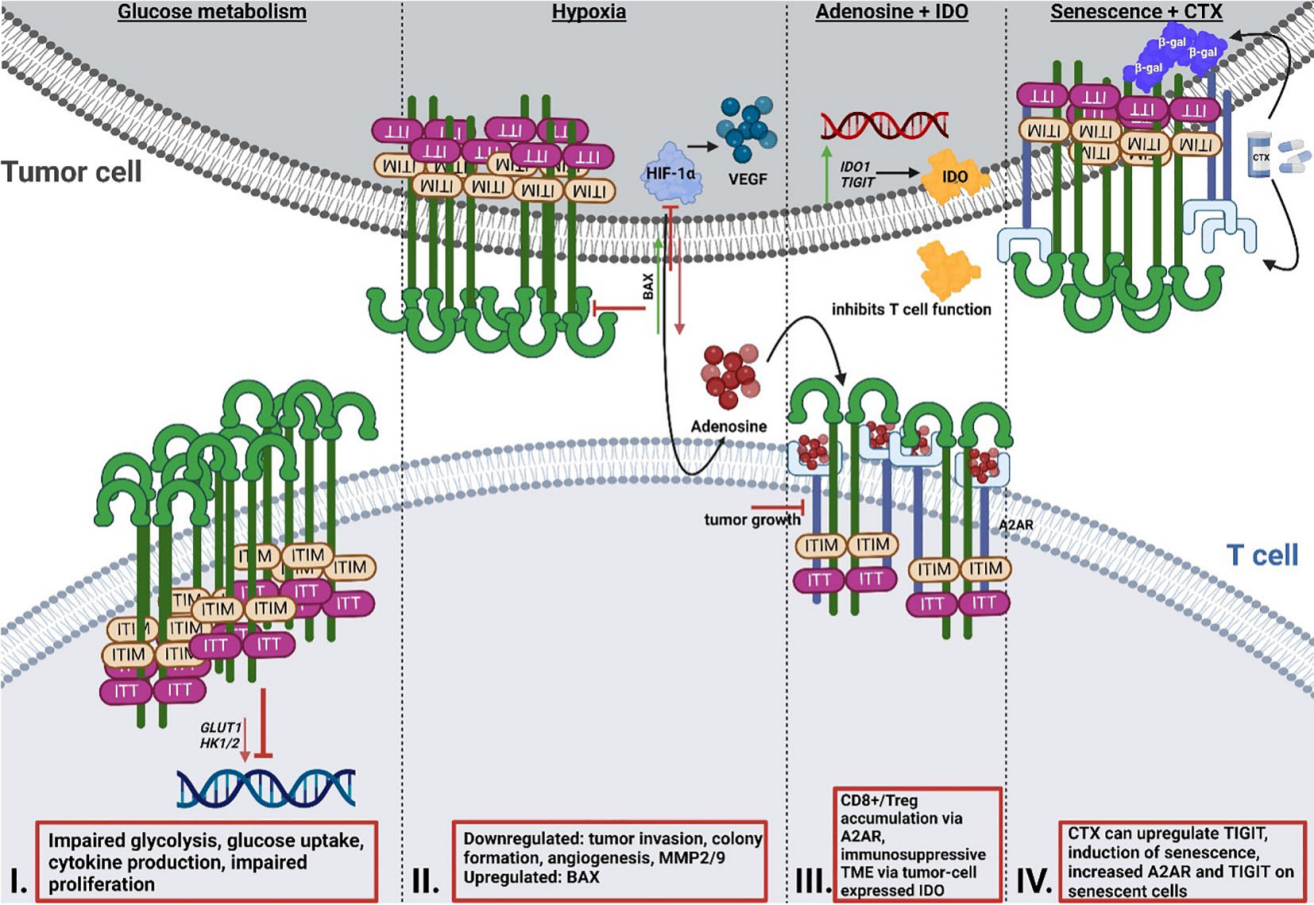
Reciprocal interaction of TIGIT signaling and T cell metabolism. **I**: Effect of TIGIT on glucose metabolism. Cancer cells inhibit T cell metabolism *via* enhancing the upregulation of TIGIT, resulting in impaired glycolysis gene expression of *GLUT1* and *HK1/2*, glucose uptake and glycolysis, and reduced proliferation. **II**: Effect of TIGIT on hypoxia and hypoxia sensing. HIF1-α regulates the expression of immune checkpoints and the expression of VEGF, which mediates tumor neovascularization. Simultaneous blocking of HIF1-α and TIGIT results in reduced tumor invasion and colony formation, as well as impaired angiogenesis and reduced MMP2/9 expression. Dual blockade leads to induction of pro-apoptotic BAX. **III**: Interaction of TIGIT and adenosine signaling and IDO. A2AR regulates the accumulation of CD8+ T cells and Tregs. Altered metabolism and hypoxia result in increased adenosine in the TME. Deletion of A2AR leads to tumor rejection in mice. IDO is highly expressed by tumor cells and generates an immunosuppressive TME. Many cancer cells overexpress *IDO1* and *TIGIT* simultaneously. **IV**: Interaction of chemotherapy and senescence and TIGIT. Chemotherapy regimens can result in the upregulation of TIGIT. A2AR is increased on the surface of senescent cells, with simultaneous upregulation of TIGIT. TIGIT, T cell immunoglobulin and ITIM domain; HK, hexokinase; GLUT1, glucose transporter 1; HIF1-α; hypoxia-inducible factor 1 alpha; MMP, matrix metalloproteinase; BAX, Bcl-2-associated X protein; IDO, indoleamine-pyrrole 2,3-dioxygenase; TME, tumor microenvironment.

A recent study by Shao et al. (92) focused on the role of TIGIT in patients with colorectal cancer and revealed that upregulated TIGIT expression in CD3^+^ T cells correlated with poor survival. In this study the authors found that T cells expressing TIGIT had impaired proliferation, cytokine production, glucose uptake, and glycolytic function. Investigations by He et al. (86) demonstrated that TIGIT^+^ CD8^+^ T cells are impaired in their effector function, allowing for the hypothesis that immune escape in gastric cancer is at least in part mediated by the upregulation of TIGIT. These TIGIT^+^ CD8^+^ T cells had significantly reduced expression of glycolysis genes, including *GLUT1* as well as (hexokinase) *HK1* and *HK2*, which resulted in impaired glucose uptake and glycolysis (**Figure 2**). Aside from cancer, another study by Calvet-Mirabent et al. (93) has shown the relevance of the connection between glucose metabolism and TIGIT as an immune checkpoint in HIV infection. In their study, the authors utilized dual blockade of PD-1 and TIGIT, as well as employed the pro-glycolytic drug Metformin, and investigated the functional properties of CD8^+^ T cells from HIV-1 patients. Significant positive correlations were observed between the increase in maximum glycolytic activity after TCR activation and the percentages of single-positive TIGIT cells, while co-expression of PD-1 and TIGIT resulted in lower glycolysis rates. Further, treatment with Metformin together with dual blockade of the two checkpoints restored cytotoxic activity of CD8^+^ T cells (93). Thus, TIGIT seems to be capable to alter T cell function *via* the inhibition of glycolysis.

#### 2.1.2 Hypoxia

Hypoxia is widely accepted to be a critical mechanism responsible for the resistance of tumor cells to radio-, chemo-, and immunotherapy (94–97). As the volume of a tumor increases, increasing numbers of cells need to be supplied with blood and oxygen, which requires additional vascularization of the tumor tissue. Without this additional supply of blood and oxygen, a state of hypoxia sets in (98). It is well established that the transcription factor hypoxia-inducible factor 1α (HIF-1α) regulates the expression of immune checkpoint proteins such as PD-L1 and CD73 (99, 100). HIF-1α is a master regulator of the cell’s response to hypoxia (101). Under normoxic conditions, the activity of HIF-1α is repressed by proteasomal degradation *via* the oxygen-dependent prolyl hydroxylase domain (PHD) and the von Hippel-Lindau (VHL) protein (102). During tumor development, HIF-1α is pivotal to the cells’ metabolic adaptation to their surroundings, as growth success under metabolic duress strongly depends upon the cell’s ability to shift from oxidative phosphorylation (OXPHOS) to the more inefficient glycolytic metabolism for ATP generation. This is accomplished by HIF-1α-regulated genes encoding enzymes for glycolysis, such as the glucose transporters *GLUT1* and *GLUT3*, *HK1*, and *HK2* as well as phosphoglycerate kinase 1 (*PGK1*) (103). HIF-1α further regulates the expression of vascular endothelial growth factor (VEGF) (104), which enables neovascularization of tumors.

So far, one study has recently addressed the synergy between TIGIT and HIF-1α (105). In this study, Fathi et al. demonstrated that simultaneous blocking of both TIGIT and HIF-1α results in a significant reduction of tumor cell invasion, decreased colony formation, and inhibited angiogenesis (105). Both matrix metalloproteinases (MMP) 2 and MMP9 as well as *VEGF* mRNA expression levels were decreased under the dual blockade. Additionally, expression of the anti-apoptotic protein B-cell lymphoma (BCL)2 was downregulated, whereas mRNA expression of the pro-apoptotic protein Bcl-2-associated X protein (BAX) was upregulated. What remains unclear is if and how, precisely, these two proteins interact with one another. Since a correlation between TIGIT and HIF1a was demonstrated by Fathi et al., further research is required to unravel the precise mechanisms of relation of the two proteins in T cells, especially when considering that HIF1a increases the expression of other immune checkpoints such as PD-L1 (11–13).

#### 2.1.3 Adenosine

Originally, adenosine receptors (ARs) were categorized into A1 or A2 ARs, depending on whether they have an inhibitory or stimulatory effect on cyclic adenosine monophosphate (cAMP) in the brain (106). Currently, ARs are categorized into four subtypes, A1, A2A, A2B and A3 (107). The majority of A2ARs are distributed in organs of the respiratory system, heart and lung, as well as in the central nervous system (CNS), and the immune system (108, 109). The adenosine receptor A2A (ADORA2) plays an important role in protecting tissues from immune-mediated damage following noninfectious inflammation, as well as in regulating the accumulation of CD8^+^ T cells and NK cells (110, 111). An altered metabolism, increased expression of CD73 as well as hypoxia (112) in the tumor can lead to higher adenosine levels in the TME (111, 113) *via* signaling through the A2A adenosine receptors (114). In this context, Ohta et al. (111) investigated the effect of A2A receptor deficiency on anti-tumor immunity mediated by CD8^+^ T cells and observed that genetic deletion of the A2A receptor results in tumor rejection in mice. Additionally, A2A receptor antagonists considerably delayed tumor growth *via* anti-tumor CD8^+^ T cells. Ohta et al. (115) have shown that immunosuppressive Tregs were induced by increased levels of extracellular adenosine, as mediated *via* A2AR stimulation. As of yet, only very few studies (116, 117) have investigated in detail the correlation between the A2A receptor and TIGIT so far. Brauneck et al. (116) investigated the correlation between the A2A receptor and TIGIT on NK cells and showed that NK-cell mediated killing of acute myeloid leukemia (AML) cells could be ameliorated by co-blockade of TIGIT and A2AR, or of TIGIT and CD39, indicating a link between the two proteins. Another study by Muhammad et al. (117) revealed that the stimulation of the A2A receptor is necessary for the emergence of TIGIT-positive Tregs in mice and that this axis is impaired in uveitis patients. This study appears to have identified a subset of TIGIT+ Tregs that are functionally dependent on the expression of the A2A receptor.

#### 2.1.4 IDO

IDO1 plays a pivotal role in the conversion of tryptophan to kynurenine (118). IDO1 is highly expressed in tumor cells and contributes to the establishment of a local immunosuppressive TME by enabling immune tolerance (119). It has been demonstrated that IDO1 inhibition induced a robust anti-tumor immune response in a mouse model when employed both as a single agent (120–127), or in combination with chemotherapeutic drugs (121, 128), highlighting the potential of IDO1 as a therapeutic target.

A recent study by Robertson et al. (129) has shown that CD8+ T cell tumor infiltrates from uveal melanoma (UM) overexpress the genes encoding for both IDO1 and TIGIT. As previously mentioned, IDO is known to limit T cell function and induce mechanisms of tolerance (130, 131). Stålhammer et al. (83) have demonstrated that not only the number of IDO^+^ cells in tumor tissues of UM appear higher than in normal choroid tissues, but that the same is true for TIGIT^+^ cells. Importantly, the number of IDO^+^ cells correlated with the number of TIGIT^+^ cells in tumor cores and full tumor sections (83). The association of TIGIT expression with IDO and PD-L1 has also been observed in the tumor core of glioblastoma (GBM) (132), underlining the necessity to further study the correlation between these proteins.

#### 2.1.5 Chemotherapy and senescence

TIGIT has recently been described as a marker for senescence due to its higher expression in aged T cells (133). The blocking of TIGIT results in improved functional capacity of senescent T cells as demonstrated by Song et al. (133), Chew et al. (134) and Kong et al. (84). The latter study also demonstrated that TIGIT expression on CD8^+^ T cells is not only elevated in acute myeloid leukemia (AML) patients, but that high TIGIT levels also correlate with primary refractory disease, as well as leukemia relapse following allogenic stem cell transplantation. TIGIT-high CD8^+^ T cells presented as functionally impaired and exhausted, whereas TIGIT blockade rescued functionality and anti-tumor response, highlighting TIGIT blockade as a potential therapeutic approach for leukemia.

Cancer treatment options in terms of chemotherapy are varied and often rely on combinatorial therapies. Some common agents used for different types of cancer are 5-Fluorouracil, an antimetabolite, DNA intercalators such as oxaliplatin and taxanes that target microtubules (135). A recent study by Davern et al. (136) revealed certain chemotherapy regimens give rise to an immune-resistant phenotype *via* the upregulation of inhibitory immune checkpoint ligands, among them TIGIT, in oesophageal adenocarcinoma (OAC). The study aimed to elucidate the effect of OAC chemotherapy approaches on the induction of a senescent-like state in cancer cells as senescent cancer cells are involved in conferring treatment resistance and promoting a microenvironment conducive to tumor growth *via* secretion of several pro-inflammatory markers, referred to as senescence-associated phenotype (SASP) (136).Using ß-galactosidase (ß-gal), an enzyme involved in the process of producing galactosylated proteins, as a marker for senescence, the authors demonstrated that the number of senescent-like cells increased significantly following chemotherapy, prompting the question whether immune checkpoints were expressed on these senescent cells or even upregulated following the treatment. The immune checkpoint TIM-3 was significantly upregulated in OE33 cells, whereas TIGIT was significantly upregulated in the SK-GT-4 cells. We know that immune checkpoints are essential for immune evasion, and if these immune checkpoints are present on senescent OAC cells, this may represent a drugable target for future therapies. Returning to another protein already addressed in this review, the adenosine receptor A2A was significantly increased on the surface of senescent-like SK-GT-4 cells, which were also shown to have increased TIGIT expression following a chemotherapy regimen. While senescent cells do have an activated glucose metabolism, they at the same time display an unbalanced lipid metabolism, which results in an altered expression of lipid metabolic enzymes, ultimately culminating in senescence induction and thereby limited functionality (137). Senescent T cells also demonstrate loss of cell surface CD28 (138–140), a protein required for lipid raft formation, IL-2 gene transcription and T cell activation. Since CD28 has also been linked to metabolic fitness of a T cell (141), the loss of this protein due to senescence can dramatically affect T cell functionality (142). Liu et al. (137) have demonstrated that the prevention of T cell senescence resulted in enhanced anti-tumor immunity, therefore maybe providing another point of potential therapeutic application.

Interestingly, TIGIT has also been shown to be intrinsically expressed in murine colorectal cell lines (143). To elucidate the functional effect of this intrinsic TIGIT, Zhou et al. (143) deleted the protein using CRISPR/Cas9 and observed that knockout resulted in significantly impaired tumor growth, together with increased IFNy secretion and cytotoxicity by NK cells, indicating that tumor cell-intrinsic TIGIT has a considerable effect on tumor growth and may present a potential therapeutic target.

### 2.2 Current status of anti-TIGIT therapeutics in clinical studies

As of August 2021, several anti-TIGIT antibodies were registered in preclinical and active clinical trials (clinicaltrials.org, anti-TIGIT). For example, two antibodies had progressed to the Phase III status (Tiragolumab (144), Ociperlimab (145)) and two were active in Phase II trials Vibostolimab, Domvanalimab) (146, 147), all of which also in combination with Atelizumab (anti-PD-L1), Pembrolizumab (anti-PD-1) and other agents. Additionally, a bispecific antibody targeting both PD-1 and TIGIT (HLX301, NCT05102214) simultaneously is under current clinical review. As discussed, TIGIT expression has been observed, among others, with PD-L1 in the tumor core (132), hinting at some kind of link between these two proteins. Currently, an anti-TIGIT candidate in combination with an anti-PD-1 antibody is being evaluated for the application for recurrent glioblastoma (148). Furthermore, increased levels of extracellular adenosine, as mediated by A2AR stimulation (114), have been shown to have a detrimental effect on anti-tumor activity (111, 115–117). Etrumadenant, an A2AR antagonist, is currently being investigated in a clinical trial in combination with Domvanalimab and Zimbrelimab (anti-PD-1) (149). It is noteworthy that the majority of the anti-TIGIT antibodies in clinical trials currently are fully human and demonstrate good tolerance by patients, also in combination with anti-PD-1 and anti-PD-L1 antibodies (150). As previously discussed in this review, TIGIT monotherapy does not result in significantly altered disease outcomes, underlining this as a potential caveat of TIGIT as a therapeutic target and highlighting the necessity for a combinatorial approach with other agents. Immune checkpoint therapy using Ipilimumab and Nivolumab as the most prominent agents has proved successful, and, taken together with the low efficacy of anti-TIGIT monotherapy, prompts the question which cohort of patients could additionally benefit from either a monotherapy or a combinatorial treatment.

### 2.3 The potential of PD-1, CTLA-4 and other negative regulators as biomarkers

Predictive biomarkers are essential to evaluate the outcome of therapeutic approaches, or at least, to provide an indication before commencement of the therapy regimen. Especially in the case of highly multifactorial diseases such as cancer and autoimmunity, such biomarkers should ideally indicate whether a monotherapy or a combinatorial approach is necessary. Here, the induction of negative regulators results in the suppression of, among other mediators, cell death mechanisms (151). Specifically these negative regulators of cell death signaling, such as heat shock proteins (HSP) (152), the Bcl-2 family (153), the PI3K/Akt/mTOR pathway (154) and others, as reviewed in detail by Razaghi et al. in (155), have found clinical application as prognostic biomarkers. In summary, negative regulators of cell death signaling appear to have great potential and present clinical application as prognostic biomarkers, raising the question whether this is also the case for the immune checkpoint proteins. When considering anti-PD-1 or anti-PD-L1 therapy, using (over-)expression of PD-L1 as biomarker appears plausible. In this context, Teng et al. (156) came up with a classification that describes PD-L1 positive tumors with infiltrating lymphocytes as a type 1 TME, proposing it to be the most likely to respond to immune checkpoint blockade. However, also PD-L1 negative tumors have been shown to be able to respond positively to antibodies targeting the PD-1/PD-L1 axis (157, 158). This consequently raises the concern that the predictive value of PD-1 and PD-L1 as biomarkers may not be optimal and universally valid across all patients, as intrapatient and even intratumor heterogeneity has been observed (159).

Other studies have hinted at the possible prognostic power of CLTA-4 expression. Here, Liu et al. (160) have demonstrated that, in some cancers, patients with higher CTLA-4 expression had a shorter overall survival than those with lower expression. However, an association between the expression levels of PD-1 and CTLA-4 and tumor-infiltrating cells exists (160). Liu et al. point out that the expression of these two immune checkpoint proteins varies across different cancers and that many cancer types demonstrate PD-1 and CTLA-4 mutations, leading to their abnormal expression, which may be used as a prognostic biomarker.

Whether TIGIT can be used in a similar manner remains to be investigated and demonstrated. Since TIGIT in its effects appears to be functionally and mechanistically tethered to other negative immune regulators such as PD-1, TIGIT alone may not prove a reliable and unambiguous prognostic biomarker. To assess the protein’s capacity of serving as a prognostic factor, large amounts of correlation data from different kinds of cancers, across different genders, ages and perhaps even ethnicities are necessary, providing information on its function and mechanistics on its own and together with other proteins that TIGIT is known to interact with. It may well be possible that a combination of factors, such as presence of PD-1, TIGIT and senescence markers will be able to form a prognostic unit of response to and success of immunotherapy in different cancers.

## 3 Discussion

While the exact role of TIGIT within the TME is still not fully elucidated, the apparent synergy between TIGIT and HIF-1α as well as PD-1 (161) does allow for the assumption that this protein does not simply have a redundant role. Based upon the literature reviewed here, blockade or targeting of TIGIT alone does not appear to have a major effect on either the progression or even curative approaches in different oncologic diseases. It is rather the combination of TIGIT blockade together with blocking of another checkpoint, such as PD-1. The fact that a synergy exists between the two is well documented and accepted to the point that several clinical trials aiming to block both proteins simultaneously are currently ongoing (162). The challenge of such a therapy, even if successful, lies in the fact that not all cancers are PD-L1 positive, thereby restricting the potential applications from the beginning. Another potential caveat is that the precise mechanism of the synergistic effects observed between the two checkpoint proteins is not fully understood, and as such it may prove difficult to design effective and individualized therapies without fully understanding the mechanistic foundations of the observed effects.

In terms of metabolism, it can be hypothesized that presence or overexpression of TIGIT poses a metabolic barrier to T cell function. Data by Gilmour et al. (163) suggest that the co-expression of TIGIT with VISTA may lead to an altered metabolic phenotype of CTL. It was been detailed in the introductory section of this review that several other immune checkpoint proteins, such as

PD-1 and CTLA-4 appear to have an effect on glycolysis of tumor cells, and thereby on the ability of immune cells to perform glycolysis due to nutrient competition within the TME. Limited nutrient ability, such as the scarcity of glucose, will lead to impaired T cell function and therefore an impaired anti-tumor response of those T cells. It is therefore crucial to further investigate the potential direct and indirect effects of TIGIT on the metabolism of T cells and other immune cells in the context of anti-tumor immunity.

It is well-known that hypoxia plays a major role in creating hostile microenvironments that are toxic to immune cells yet conducive to tumor growth. So far, only one study has investigated the direct interaction between HIF-1α and TIGIT. It remains an open question whether a potential three-way synergy might exist between blocking not only TIGIT and PD-1, but also HIF-1α. Along this line, it would be important to assess whether a co-blockade of TIGIT and HIF-1α is as effective as the blockade of TIGIT and PD-1 as a therapeutic possibility for those cancers which are not PD-L1 positive.

The interplay between TIGIT and adenosine as well as the A2A receptor makes for another interesting point of further investigation. The genetic deletion of the A2A receptor in mice resulted in tumor rejection (162), allowing for the hypothesis that some connection may also exist between these proteins. Additionally, it is known that hypoxia leads to higher adenosine levels in the TME, prompting the question whether the TIGIT-A2AR-HIF-1α axis could provide another possible three-way blockade for therapeutic purposes. The A2A receptor was additionally observed to be upregulated on the surface senescent cancer cells, which at the same time showed increased TIGIT expression following some chemotherapy regimens.

The potential of TIGIT expression as a biomarker has been suggested, although for this, larger association studies are needed. Future experiments should aim to elucidate the connection between TIGIT and other immune checkpoints, particularly those involved in the immune response against cancers which do not express PD-L1, as well as the interplay with HIF-1α and the A2A receptor. Perhaps this will lead to a better understanding of the exact mechanisms governing the synergistic inhibitory effects of combination treatments. Taken together, TIGIT appears to have a therapeutic potential, especially in the context of combinatorial therapies and alleviating the metabolic barrier that immune checkpoint proteins are able to pose, that should not be overlooked and disregarded for further research, both of basic and translational nature.

## Author contributions

NJ-N and SK designed the study. NJ-N performed literature search. NJ-N and SK drafted the manuscript. NJ-N designed **Figures 1** and **2.** RB-L, KB, MM-F, MB, BS and DM provided critical input throughout the work and corrected the manuscript. SK and BS together with DM supervised the work. All authors contributed to the article and approved the submitted version.
